# Characterization of skin adverse events associated with cetuximab: real-world insights from the two global pharmacovigilance databases of FAERS and VigiAccess

**DOI:** 10.3389/fonc.2026.1768984

**Published:** 2026-02-16

**Authors:** Chenwen Li, Yifei Gao, Min Guang, Huajuan Wu, Yunfei Li, Yaqing Lu, Yan Wei, Xueli Li, Li Yang

**Affiliations:** Department of Dermatology, Henan Provincial People’s Hospital, Henan University People’s Hospital, Zhengzhou, China

**Keywords:** cetuximab, disproportionality analysis, FAERS, skin adverse events, VigiAccess

## Abstract

**Background:**

Cetuximab, an IgG1 monoclonal antibody targeting the epidermal growth factor receptor (EGFR), is widely used in metastatic colorectal cancer and other solid tumors. Cutaneous adverse events (AEs) are among its most frequent toxicities, but real-world evidence on their spectrum and risk profile remains limited.

**Methods:**

We conducted a pharmacovigilance study using spontaneous reports from the FDA Adverse Event Reporting System (FAERS) and the World Health Organization VigiAccess database, from database inception to 2025. Reports in which cetuximab was recorded as the primary suspected drug were retrieved. After deduplication, AEs were coded using MedDRA preferred terms (PTs) and system organ classes. Descriptive analyses were performed, and disproportionality was evaluated using reporting odds ratio (ROR), proportional reporting ratio (PRR), empirical Bayes geometric mean (EBGM), and information component (IC). Subgroup analyses by age and sex were conducted using FAERS data.

**Results:**

A total of 69,588 reports were identified, including 20,862 from FAERS and 48,726 from VigiAccess. Most reports involved male patients and those aged 45–64 or ≥65 years. Skin and subcutaneous tissue disorders showed significant disproportionality signals for cetuximab (FAERS: ROR 2.58, 95% CI 2.52–2.65; VigiAccess: ROR 2.73, 95% CI 2.69–2.77). The most frequently reported skin-related PTs were rash, dermatitis acneiform, and erythema in FAERS, and rash, acne, and dermatitis acneiform in VigiAccess. The strongest signals were observed for dermatitis acneiform, nail bed inflammation, and rash follicular in FAERS, and for dermatitis acneiform, nail cuticle fissure, and nail fold inflammation in VigiAccess. Subgroup analyses indicated that skin AEs were more commonly reported in patients older than 45 years and in males.

**Conclusions:**

Real-world pharmacovigilance data from two independent international databases demonstrate consistent and strong disproportionality signals for cetuximab-associated cutaneous AEs. Common events such as rash and dermatitis acneiform, as well as several less frequently described reactions, warrant heightened clinical vigilance, especially in older and male patients. Prospective and mechanistic studies are needed to confirm these associations and to refine strategies for preventing and managing cetuximab-induced skin toxicity.

## 1 Introduction

Colorectal cancer (CRC) remains one of the most common malignancies worldwide. According to the most recent global cancer statistics from GLOBOCAN 2022, there were approximately 1.9 million new CRC cases and 0.9 million CRC-related deaths globally, making CRC the third most frequently diagnosed cancer and the second leading cause of cancer death worldwide. By 2040, global CRC cases are projected to increase further, potentially exceeding 3.2 million new cases annually (1). Historically, the identification of CRC has relied heavily on histopathological analysis (2), while the main clinical symptoms include alterations in bowel patterns, abdominal pain, and rectal hemorrhage (3). Notably, 20% of patients present with metastatic disease at the time of their initial diagnosis. Recently, significant advancements have been made in improving the prognosis for metastatic colorectal cancer (mCRC), largely due to the introduction of new molecular targeted therapies. Notably, agents targeting epidermal growth factor receptor (EGFR) have demonstrated considerable clinical efficacy (4). Elevated levels of EGFR are observed in various epithelial tumors, including those of the prostate, breast, head and neck, as well as gastrointestinal cancers (4–7). In CRC, the expression of EGFR is significantly higher than that in normal colorectal mucosa (8, 9). In 2004, the U.S. Food and Drug Administration approved cetuximab, an IgG1 monoclonal antibody that specifically targets EGFR, for the treatment of CRC.

Cetuximab is a chimeric monoclonal antibody composed of both human and murine components that competitively binds to the extracellular domain of EGFR (10). This binding inhibits dimerization, thereby disrupting intracellular signaling pathways. Consequently, several effects occur, including the inhibition of cancer cell proliferation, enhancement of apoptosis, and reduction in the production of matrix metalloproteinases (11). In recent years, significant advancements have been made in the management of mCRC through the use of cetuximab in conjunction with chemotherapy (12). Moreover, research has explored the use of cetuximab in treating various malignant tumors, including breast cancer, pancreatic cancer, head and neck squamous cell carcinoma (13), and esophageal squamous cell carcinoma (14). Consequently, the widespread application of cetuximab underscores the necessity of understanding its associated adverse effects, which may include mucosal inflammation, rash, myelosuppression, gastrointestinal issues (such as nausea, vomiting, diarrhea, and constipation), and interstitial pneumonia (15–17). While clinical studies (18) have indicated that skin reactions are the most common adverse effects, there remains a significant lack of comprehensive real-world research to thoroughly evaluate the cutaneous safety profile of cetuximab.

The FDA Adverse Event Reporting System (FAERS) and VigiAccess are two primary databases for spontaneous reporting, providing essential real-world evidence for pharmacovigilance in the post-marketing phase (19). Managed by the U.S. Food and Drug Administration, FAERS plays a critical role in identifying safety signals related to adverse events (AEs) associated with approved pharmaceuticals and biological products (20). VigiAccess, developed and maintained by the Uppsala Monitoring Centre on behalf of the World Health Organization (WHO), serves as a global database for suspected adverse drug reactions, facilitating the timely identification of safety issues across diverse populations and therapeutic contexts (21, 22). As cetuximab becomes more widely used in clinical settings, it is essential to comprehend its adverse effect profile in detail, especially regarding cutaneous toxicity, to improve patient safety and guide treatment approaches. This study employs two reputable datasets, FAERS and VigiAccess, to assess the potential for cutaneous adverse events linked to cetuximab, with the goal of enhancing its safety profile and offering evidence-based recommendations for healthcare providers and policymakers in the management of these occurrences.

## 2 Materials and methods

### 2.1 Data source

The FAERS and VigiAccess databases, which are accessible to the public, gather worldwide reports concerning adverse events (AEs), medication errors, and complaints regarding product quality from both healthcare providers and consumers. These two interconnected systems function as vital tools for overseeing post-market drug safety and facilitate thorough assessments of the risks linked to medications.

This study included human participants and adhered to the applicable regulations and policies of the institution. As a result, ethical clearance was not necessary. In line with national and institutional protocols, it was not obligatory to acquire written informed consent from participants or their legal guardians or family members for involvement.

**Figure 1:**
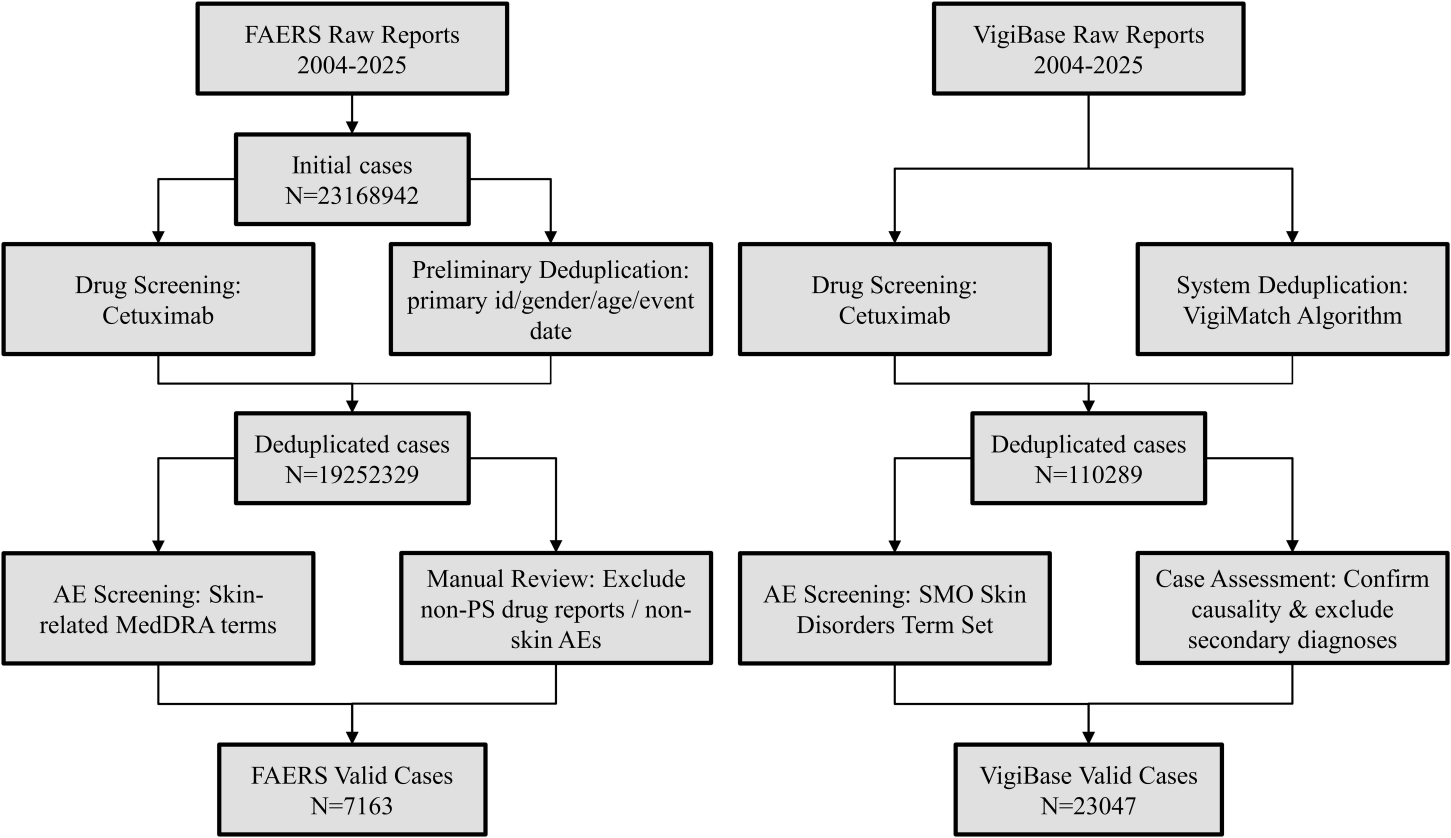
Flow diagram for the selection of skin AEs with Cetuximab from the FAERS and VigiAccess databases.

In this study, adverse event (AE) reports related to cetuximab were gathered from the FAERS and VigiAccess databases. The data retrieval period for the study encompassed the entirety of each database’s available records, extending from their inception until the second quarter of 2025 for the FAERS database and up to December 2024 for the VigiAccess database. This comprehensive timeframe ensures a robust analysis of the data trends and patterns relevant to the study objectives. The gathered information encompassed patient demographic details, including age and gender, in addition to the classification of AEs based on system organ class (SOC). To guarantee the data’s precision and dependability, a thorough deduplication procedure was carried out to remove duplicate entries, thereby retaining only complete and pertinent records for further analysis. The overall workflow for data processing is illustrated in **Figure 1**.

### 2.2 Data extraction, processing and AE description methodology

For FAERS, raw quarterly ASCII data from Q1–2004 to Q2–2025 were downloaded from the FDA website and processed using SAS (version 9.4). Drug names were standardized according to the WHO Drug Dictionary, and reports were identified using the generic name “cetuximab,” with only cases in which cetuximab was recorded as the primary suspected (PS) drug retained. Deduplication was performed in accordance with FDA recommendations by consolidating reports with the same CASEID and retaining the record with the most recent FDA_DT; when both CASEID and FDA_DT were identical, the report with the largest PRIMARYID was kept, and deleted cases listed in FDA quarterly deletion files were excluded. All adverse events were recoded using the latest version of MedDRA and mapped to Preferred Terms (PTs) and System Organ Classes (SOCs), after which skin-related adverse events were identified based on MedDRA skin and subcutaneous tissue disorder terms. Reports not primarily related to skin adverse events were further excluded through manual review.

For VigiAccess, data were retrieved from the WHO VigiBase via the VigiAccess platform using automated queries based on WHO Drug Dictionary BASENAME entries, and duplicate reports were handled by the VigiMatch algorithm, the standard WHO–Uppsala Monitoring Centre method for identifying duplicate individual case safety reports. Skin adverse events were identified using the Standardized MedDRA Query (SMQ) for skin and subcutaneous tissue disorders (23, 24). After applying all filtering and deduplication steps, the remaining valid cases from both databases were included in subsequent disproportionality analyses.

### 2.3 Signal mining methodology

Disproportionality analysis, recognized as a prominent method in the field of post-marketing pharmacovigilance, was employed as the primary strategy for signal detection in this investigation. Four conventional metrics were applied: Reporting Odds Ratio (ROR), Proportional Reporting Ratio (PRR), Empirical Bayes Geometric Mean (EBGM), and Bayesian Confidence Propagation Neural Network (BCPNN). ROR was used as the primary signal detection metric, while PRR, EBGM, and IC were applied as complementary methods for robustness assessment (**Supplementary Table S1**). These additional algorithms aided in uncovering possible safety signals related to skin adverse events connected to cetuximab. Data extraction and statistical analyses were conducted independently by two investigators utilizing IBM^®^ SPSS^®^ Statistics (version 27.0) and R software (version 4.3), ensuring the reliability and reproducibility of the findings.

## 3 Results

### 3.1 Descriptive analysis

This study investigated adverse reactions associated with cetuximab using data from the FAERS and VigiAccess databases. A total of 69,588 AE reports were identified, including 20,862 from FAERS and 48,726 from VigiAccess (**Table 1**). In the FAERS database, 5,670 reports (27.18%) were submitted by female patients and 12,336 reports (59.13%) by male patients. Similarly, in the VigiAccess database, 15,013 reports (30.81%) were associated with female patients and 30,483 reports (62.56%) with male patients, indicating a slightly higher reporting frequency of AEs among males in both databases.

**Table 1:** Reported characteristics of adverse events associated with Cetuximab.

Characteristics	FAERS (n=20862)	Vigiaccess (n=48726)
Gender, n (%)		
Female	5670 (27.18)	15013 (30.81)
Male	12336 (59.13)	30483 (62.56)
Unknown	2856 (13.69)	3230 (6.63)
Age (years), n (%)		
<18 (%)	16 (0.08)	49 (0.11)
18-44 (%)	922 (4.42)	3165 (6.5)
45-64 (%)	6942 (33.28)	17403 (35.72)
≥65 (%)	6685 (32.04)	16019 (32.88)
Not Specified (%)	6297 (30.18)	12090 (24.81)
Reporting country (%)		
Americas (%)	10313 (49.44)	23880 (49.01)
Europe (%)	7835 (37.56)	11985 (24.6)
Asia (%)	1407 (6.74)	11649 (23.91)
Oceania (%)	44 (0.21)	473 (0.97)
Africa (%)	11 (0.05)	739 (1.52)
Reports n (%)		
Consumer (%)	11903 (57.06)	
Other health-professional (%)	5024 (24.08)	
Physician (%)	2288 (10.97)	
Pharmacist (%)	1252 (6.00)	
Lawyer (%)	2 (0.01)	
Not Specified (%)	393 (1.88)	
Outcome n (%)		
Hospitalization-Initial or Prolonged (%)	7915 (37.94)	
Death (%)	2307 (11.06)	
Life-Threatening (%)	1704 (8.17)	
Disability (%)	360 (1.73)	
Required Intervention to Prevent Permanent Impairment/Damage (%)	119 (0.57)	
Congenital Anomaly (%)	3 (0.01)	
Other (%)	8331 (39.93)	

Concerning the distribution of ages in FAERS, the highest percentage of adverse event reports related to cetuximab was found in individuals aged 45–64 years (n = 6,942; 33.28%). This was followed by patients aged 65 years and older (n = 6,685; 32.04%) and those aged 18–44 years (n = 922; 4.42%). A comparable trend appeared in VigiAccess, where the predominant number of reports came from patients aged 45–64 years (n = 17,403; 35.72%), followed by individuals aged 65 years and above (n = 16,019; 32.88%) and those in the 18–44 age range (n = 3,165; 6.50%).

**Figure 2:**
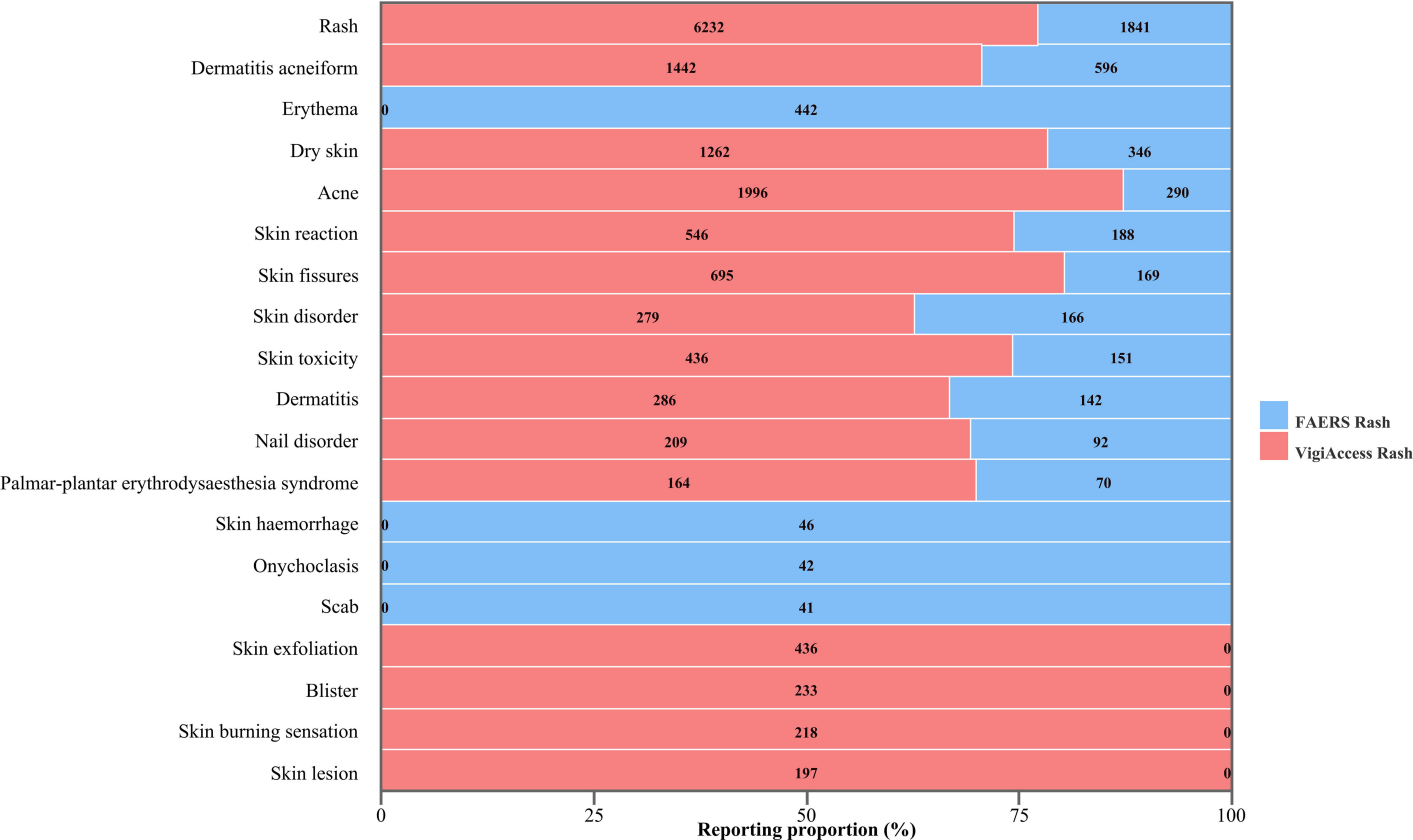
The annual number of reported AEs associated with cetuximab in the FAERS and VigiAccess databases.

Geographically, in FAERS, the majority of reports originated from the Americas (n = 10,313; 49.44%), followed by Europe (n = 7,835; 37.56%). Likewise, in VigiAccess, most reports were from the Americas (n = 23,880; 49.01%), with additional contributions from Europe (n = 11,985; 24.60%) and Asia (n = 11,649; 23.91%). Temporal analysis revealed that the peak reporting years were 2013 for FAERS (1,552 cases) and 2024 for VigiAccess (5,124 cases), as shown in **Figure 2**.

In the FAERS database, the largest proportion of cetuximab-related AE reports was submitted by consumers (n = 11,903; 57.06%), followed by other health professionals (n = 5,024; 24.08%), physicians (n = 2,288; 10.97%), and pharmacists (n = 1,252; 6.00%). Among reported outcomes, hospitalization occurred in 7,915 cases (37.94%), and significant medical events were recorded in 8,331 reports (39.93%). Notably, 2,307 fatal cases (11.06%) were reported, indicating the potential severity of cetuximab-associated AEs.

### 3.2 skin AEs associated with cetuximab from two databases

**Figure 3:**
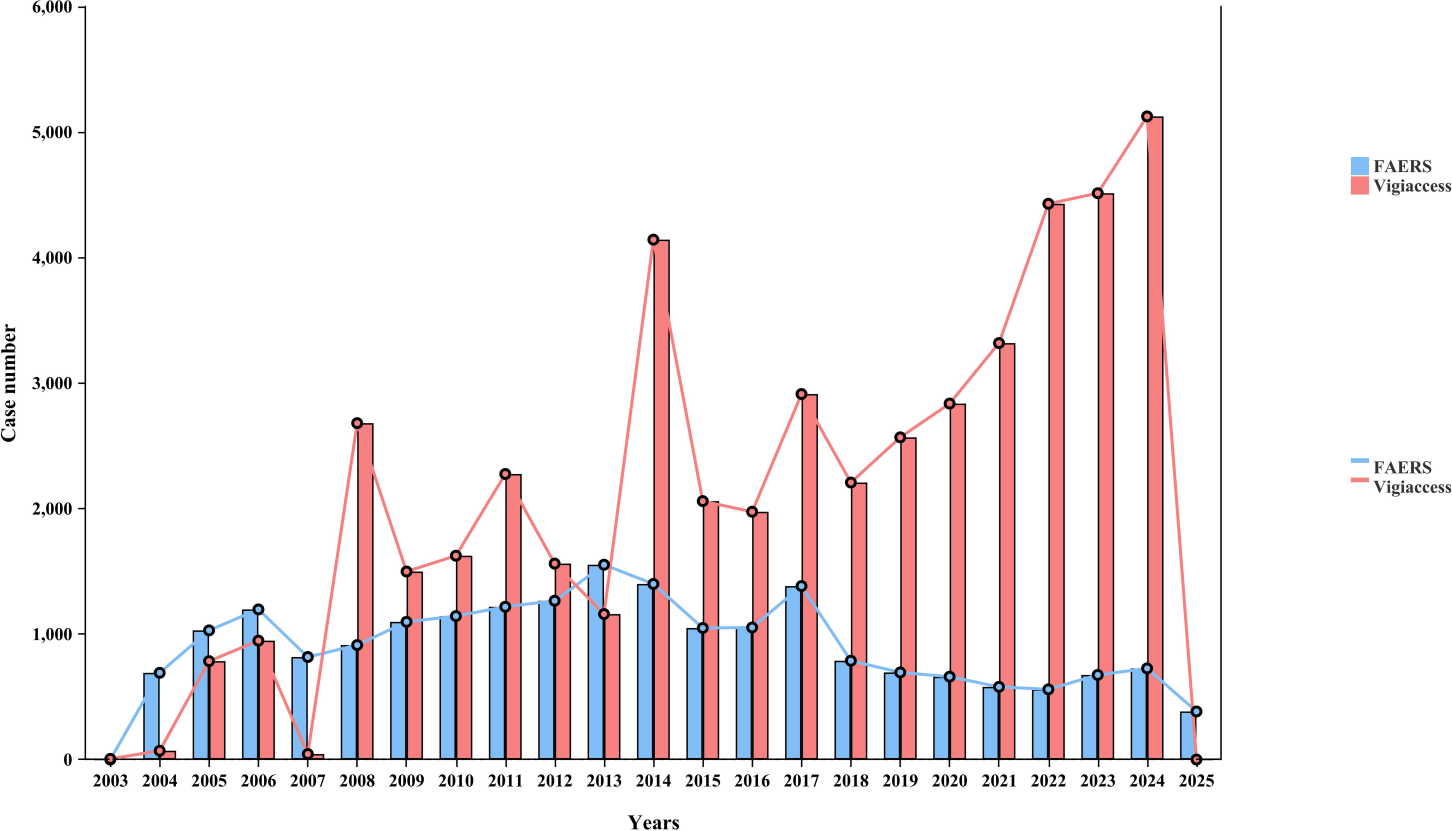
Distribution of the top 15 cetuximab-associated skin-related PTs with positive signals ranked by reporting frequency in the FAERS and VigiAccess databases.

**Figure 4:**
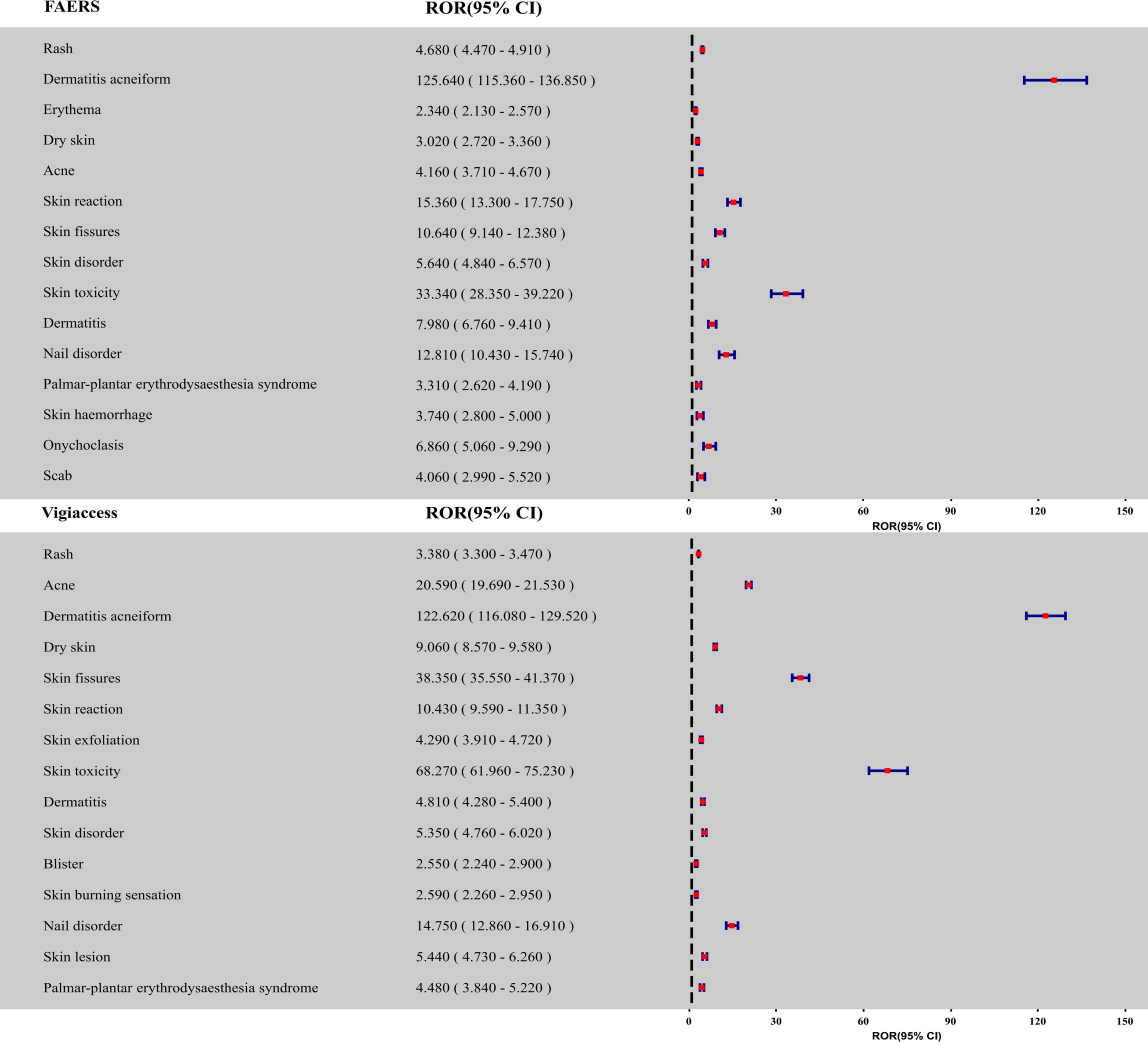
Forest plot of RORs for cetuximab-associated skin adverse events in FAERS and VigiAccess.

The assessment of skin AEs related to cetuximab, as outlined in **Table 2**, revealed consistent results in both datasets. The ROR were found to be 2.58 (95% confidence interval [CI]: 2.52, 2.65) for the FAERS database and 2.73 (95% CI: 2.69, 2.77) for VigiAccess, showing a significant correlation between cetuximab and skin AEs. To enhance the understanding of the skin toxicity spectrum, a *post hoc* analysis was conducted at the PT level using combined data from both databases. **Table 3** and **Figure 3** display the 15 most common skin-related PTs associated with cetuximab, along with their respective RORs shown in **Figure 4**. Within the FAERS database, the PTs most frequently reported were rash (n = 1841), acneiform dermatitis (n = 596), and erythema (n = 442). In contrast, the VigiAccess database revealed the most prevalent PTs as rash (n = 6232), acne (n = 1996), and acneiform dermatitis (n = 1442). **Table 3** illustrates a strong correlation between the two data sources. Additionally, the disproportionality analysis identified notable safety signals for various skin adverse events linked to cetuximab, with comprehensive details available in **Supplementary Table S1**. In the FAERS database, the highest reporting odds ratios (RORs) were noted for dermatitis acneiform (ROR = 125.64; 95% CI: 115.36, 136.85), inflammation of nail beds (ROR = 66.70; 95% CI: 40.84, 108.94), and follicular rash (ROR = 53.29; 95% CI: 24.93, 113.93). Likewise, in VigiAccess, dermatitis acneiform (ROR = 122.62; 95% CI: 116.08, 129.52), fissures in nail cuticles (ROR = 85.96; 95% CI: 39.80, 185.67), and inflammation of nail folds (ROR = 74.97; 95% CI: 27.20, 206.63) showed significantly elevated signal values. These results highlight the essential importance of monitoring skin toxicity in patients undergoing treatment with cetuximab, especially concerning nail fold inflammation and dermatitis acneiform.

**Table 2:** Signal detection of Cetuximab-associated skin disorder adverse events from two databases.

Cetuximab	The report number	ROR (95%CI)	PRR (χ2)	EBGM (EBGM05)	IC (IC025)
FAERS	7163	2.58 (2.52,2.65)	2.38 (6030.76)	2.37 (2.32)	1.25 (1.21)
Vigiaccess	23047	2.73 (2.69,2.77)	2.37 (19955.6)	2.37 (2.33)	1.24 (1.22)

PRR, the proportional reporting ratio; ROR, the reporting odds ratio; IC, the information component; EBGM, the empirical Bayes geometric mean; CI, confidence interval; 95%CI, two-sided for ROR; **χ**^2^, chi-squared; IC025 and EBGM05 lower one-sided for IC, and EBGM.

**Table 3:** Disproportionality analysis of cetuximab-associated skin adverse events in FAERS and VigiAccess.

Cetuximab	The report number	ROR (95%CI)	PRR (χ^2^)	EBGM (EBGM05)	IC (IC025)
FAERS					
Rash	1841	4.68 (4.47,4.91)	4.56 (5131.68)	4.54 (4.34)	2.18 (2.11)
Dermatitis acneiform	596	125.64 (115.36,136.85)	124.31 (65039.3)	111.00 (101.91)	6.79 (6.43)
Erythema	442	2.34 (2.13,2.57)	2.33 (336.20)	2.33 (2.12)	1.22 (1.08)
Dry skin	346	3.02 (2.72,3.36)	3.01 (463.26)	3.00 (2.70)	1.59 (1.42)
Acne	290	4.16 (3.71,4.67)	4.15 (690.51)	4.13 (3.68)	2.05 (1.86)
Skin reaction	188	15.36 (13.30,17.75)	15.31 (2479.02)	15.10 (13.08)	3.92 (3.60)
Skin fissures	169	10.64 (9.14,12.38)	10.61 (1456.01)	10.51 (9.03)	3.39 (3.09)
Skin disorder	166	5.64 (4.84,6.57)	5.63 (628.76)	5.60 (4.81)	2.49 (2.22)
Skin toxicity	151	33.34 (28.35,39.22)	33.26 (4576.43)	32.24 (27.41)	5.01 (4.50)
Dermatitis	142	7.98 (6.76,9.41)	7.96 (857.49)	7.90 (6.70)	2.98 (2.67)
Nail disorder	92	12.81 (10.43,15.74)	12.79 (988.14)	12.65 (10.30)	3.66 (3.19)
Palmar-plantar erythrodysaesthesia syndrome	70	3.31 (2.62,4.19)	3.31 (112.32)	3.30 (2.61)	1.72 (1.33)
Skin haemorrhage	46	3.74 (2.80,5.00)	3.74 (91.95)	3.73 (2.79)	1.90 (1.40)
Onychoclasis	42	6.86 (5.06,9.29)	6.85 (208.54)	6.81 (5.03)	2.77 (2.14)
Scab	41	4.06 (2.99,5.52)	4.06 (94.21)	4.05 (2.98)	2.02 (1.47)
Vigiaccess					
Rash	6232	3.38 (3.30,3.47)	3.25 (9837.39)	3.24 (3.16)	1.70 (1.66)
Acne	1996	20.59 (19.69,21.53)	20.23 (35845.9)	19.88 (19.01)	4.31 (4.23)
Dermatitis acneiform	1442	122.62 (116.08,129.52)	121.03 (154199)	108.81 (103.01)	6.77 (6.58)
Dry skin	1262	9.06 (8.57,9.58)	8.97 (8872.33)	8.90 (8.42)	3.15 (3.06)
Skin fissures	695	38.35 (35.55,41.37)	38.11 (24256.1)	36.84 (34.14)	5.20 (5.02)
Skin reaction	546	10.43 (9.59,11.35)	10.39 (4589.13)	10.30 (9.46)	3.36 (3.22)
Skin exfoliation	436	4.29 (3.91,4.72)	4.28 (1092.88)	4.27 (3.88)	2.09 (1.94)
Skin toxicity	436	68.27 (61.96,75.23)	68.01 (27065.1)	64.00 (58.08)	6.00 (5.66)
Dermatitis	286	4.81 (4.28,5.40)	4.80 (856.24)	4.78 (4.26)	2.26 (2.07)
Skin disorder	279	5.35 (4.76,6.02)	5.34 (980.66)	5.32 (4.73)	2.41 (2.22)
Blister	233	2.55 (2.24,2.90)	2.55 (218.38)	2.54 (2.24)	1.35 (1.15)
Skin burning sensation	218	2.59 (2.26,2.95)	2.58 (211.02)	2.58 (2.26)	1.37 (1.16)
Nail disorder	209	14.75 (12.86,16.91)	14.72 (2637.20)	14.54 (12.68)	3.86 (3.57)
Skin lesion	197	5.44 (4.73,6.26)	5.43 (708.99)	5.41 (4.70)	2.44 (2.20)
Palmar-plantar erythrodysaesthesia syndrome	164	4.48 (3.84,5.22)	4.47 (440.61)	4.46 (3.82)	2.16 (1.90)

PRR, the proportional reporting ratio; ROR, the reporting odds ratio; IC, the information component; EBGM, the empirical Bayes geometric mean; CI, confidence interval; 95%CI, two-sided for ROR, **χ**^2^, chi-squared; IC025 and EBGM05 lower one-sided for IC, and EBGM.

### 3.3 Subgroup analysis

**Figure 5:**
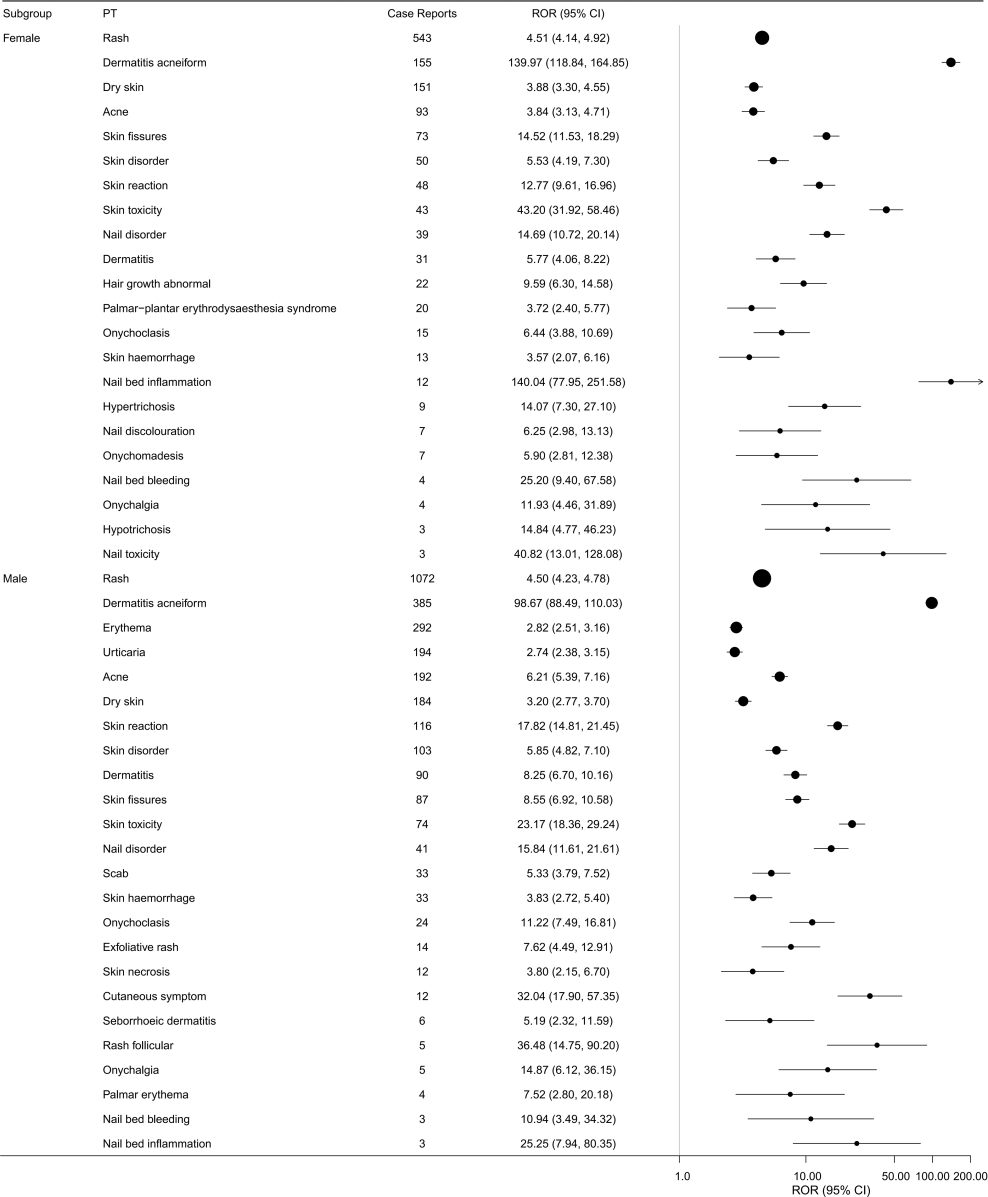
Cetuximab-associated skin-related PTs ranked by the frequency of positive signals across different age groups.

**Figure 6:**
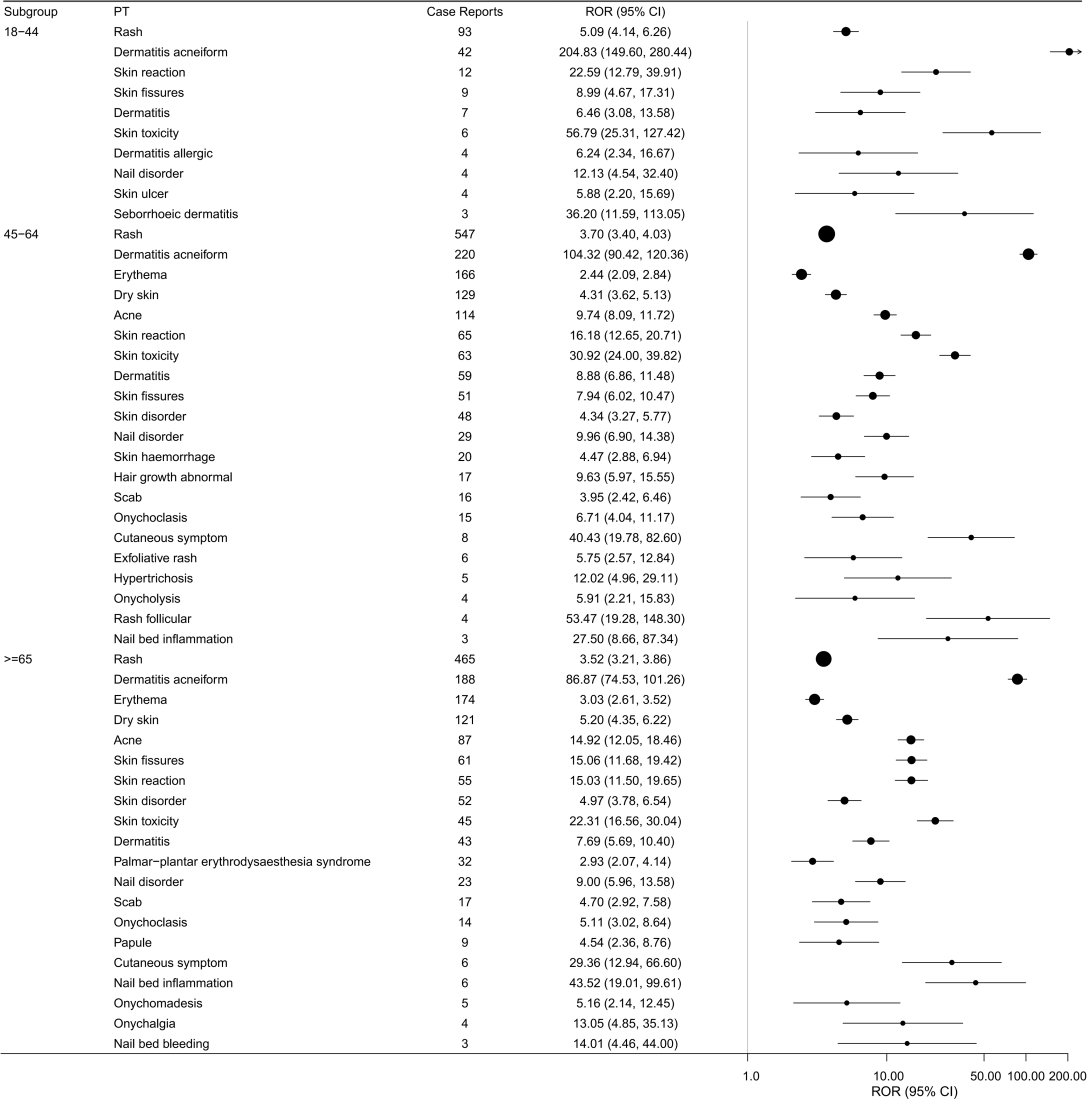
Cetuximab-associated skin-related PTs ranked by the frequency of positive signals across different gender groups.

To evaluate individual traits and potential risk factors linked to skin-related adverse reactions induced by cetuximab, subgroup analyses were performed according to age (see **Figure 5**) and gender (refer to **Figure 6**), utilizing all adverse events documented in the FAERS database. Among the various subgroups analyzed, rash emerged as the most commonly reported skin-related adverse event. The analysis stratified by age revealed that patients over 45 years old experienced skin-related adverse reactions more frequently. Moreover, the gender-based analysis indicated a greater reporting frequency among male patients. Overall, these results suggest that skin adverse events associated with cetuximab are reported more often in older individuals and in males.

## 4 Discussion

At present, there is a notable lack of research centered on pharmacovigilance regarding skin AEs related to cetuximab. This study is, to our knowledge, the first in-depth evaluation of skin toxicity linked to cetuximab in a real-world context, leveraging data sourced from the FAERS and VigiAccess databases. Through performing disproportionality analyses across two distinct international reporting systems, we identified strong and consistent safety signals for multiple skin-related PTs, including rash, nail fold inflammation, and acneiform dermatitis. Furthermore, our subgroup analysis indicated that these AEs primarily impact older adults (those over 45 years) and males, highlighting the importance of prompt clinical monitoring. These findings offer fresh insights into the specific skin safety profile of cetuximab and bear significant consequences for pharmacovigilance practices and strategies for managing clinical risks.

It has been noted that there is a positive correlation between the severity of rash and the effectiveness of its treatment (25). Regulatory authorities in North America and Europe have granted approval for cetuximab for the treatment of advanced CRC (12) and head and neck squamous cell carcinoma (26). Nevertheless, the prevalence of these dermatological effects has not been comprehensively assessed in post-marketing surveillance. This research seeks to fill this void by investigating the association between cetuximab and skin AEs through data obtained from two large pharmacovigilance databases. Our findings highlight the importance of establishing routine skin monitoring in the clinical management of patients receiving cetuximab.

**Table 1** illustrates that AEs from cetuximab were reported more frequently in male patients (59.13% in FAERS; 62.56% in VigiAccess) compared to female patients (27.18% in FAERS; 30.81% in VigiAccess). This disparity between sexes may be associated with the higher incidence and mortality rates observed in males diagnosed with CRC (27), leading to increased exposure to cetuximab within this demographic.

Our analysis of the FAERS and VigiAccess databases indicates a notable correlation between cetuximab and a range of inflammatory skin reactions, such as acneiform dermatitis, erythema, and dermatitis. In addition, we identified robust disproportionality signals for less frequently reported conditions like exfoliative dermatitis, seborrheic dermatitis, and allergic dermatitis. Moreover, both databases show that rashes are the most common issues impacting the skin and subcutaneous tissues. These research results align with those from a prior multicenter investigation into cetuximab’s efficacy and toxicity for treating head and neck squamous cell carcinoma (HNSCC), which found that skin rash occurred as the most commonly reported adverse event linked to this therapy, with an incidence of 49% (28). Although skin and subcutaneous tissue disorders were the most commonly reported adverse effects, only a small fraction of patients needed adjustments to their dosages because of serious symptoms. Nevertheless, even mild to moderate skin issues can lead to ongoing itching, worsening of the skin, or a higher likelihood of infections, which can greatly affect the quality of life for patients (29). Expert recommendations suggest that diffuse rashes or worsened acneiform dermatitis might indicate significant mucocutaneous inflammatory reactions. This highlights the importance of careful monitoring for possible secondary infections or scarring. Patients should be encouraged to avoid scratching the affected regions, steer clear of irritating skincare products, and quickly communicate any new or deteriorating skin issues (30). These findings underscore the vital necessity of diligently observing the dermatological condition of patients, commencing early interventions, and delivering focused health education during the entire course of cetuximab treatment.

Skin and subcutaneous tissue disorders linked to cetuximab might occur due to the disturbance of signaling in the EGFR pathway. Such disturbances can lead to various effects, which manifest as the typical clinical symptoms of skin toxicity caused by EGFR inhibitors. The EGFR is essential for the proper differentiation and development of epidermal keratinocytes, as it aids in epidermal growth, hinders differentiation, offers protection from ultraviolet (UV) radiation damage, lessens inflammation, and supports wound healing (31, 32). A study employing immunohistochemistry in the context of pharmacological inhibition of EGFR has demonstrated notable changes in the expression of crucial markers within the skin. The inhibition of EGFR resulted in the removal of cells that showed phosphorylated EGFR, along with diminished MAPK expression levels (33, 34). This modification subsequently impacted the behavior of keratinocytes, affecting aspects such as proliferation, differentiation, migration, and adhesion. This led to a range of skin responses, including rash, erythema, and dryness. Up until now, the majority of investigations into the mechanisms that drive acneiform eruptions triggered by EGFR inhibitors have primarily focused on their influence on keratinocyte proliferation and differentiation, ultimately leading to secondary inflammation. Nevertheless, a recent study shows that therapies aimed at targeting EGFR may have a more immediate effect on the immune response by reducing the inhibition of skin chemokine production, thereby promoting the chemotaxis and infiltration of leukocytes into the skin. This indicates that the role of skin EGFR is associated with the inhibition of chemokine synthesis (35). In summary, these findings support a biologically plausible mechanism underlying the cutaneous adverse effects associated with cetuximab, driven by interference in EGFR pathway signaling.

Given the important function of EGFR signaling in the skin, the considerable physical and psychological distress that may occur could require modifications in the dosage of anticancer drugs or possibly their cessation. We suggest that active skin monitoring be included in the clinical protocols for patients receiving treatment with cetuximab. Specialists from Italy concur that a team-based management strategy can alleviate skin-related toxicities, lower the incidence of severe symptoms, boost patient compliance, avert changes in prescribed treatments (such as radiotherapy and/or EGFR inhibitors), and ultimately enhance overall results (36). A proactive and cooperative approach to preventing and managing skin toxicity can greatly decrease the occurrence of serious symptoms, improve patient comfort during treatments, enhance the overall quality of life, and optimize the therapeutic advantages of EGFR-targeted monoclonal antibodies by reducing the necessity for dosage adjustments or early termination of therapy. Additionally, specifically trained nursing personnel can proficiently handle minor skin reactions. As a result, in the consensus guidelines focused on managing skin toxicity due to radiation in head and neck cancer, the involvement of nurses is vital for situations identified as low-grade (37). Recent studies have highlighted the potential of topical vitamin K3 (menadione) in inhibiting EGFR phosphatase, thereby restoring disrupted EGFR-mediated signaling in the skin following systemic administration of the EGFR inhibitor cetuximab (38). Although additional rigorously designed prospective research is necessary to confirm best practices, instruments like this algorithm can assist healthcare providers and inform patients, thereby improving the efficacy of cetuximab treatment. With the growing application of these drugs in early-stage and adjuvant therapy contexts, developing evidence-backed methods to manage cutaneous toxicity will become ever more important.

Despite the strengths of this study, several limitations inherent to spontaneous reporting systems should be acknowledged. First, both FAERS and VigiAccess lack reliable denominator data, such as the total number of patients exposed to cetuximab, which precludes the estimation of true incidence rates or absolute risks. Second, underreporting and reporting bias are well-recognized issues in pharmacovigilance databases, particularly for mild or transient adverse events, which may lead to an underestimation of the overall burden or an overrepresentation of well-known or expected reactions. Third, although FAERS provides outcome-related information (e.g., hospitalization, life-threatening events, or death), neither FAERS nor VigiAccess systematically captures standardized clinical severity grading, such as Common Terminology Criteria for Adverse Events (CTCAE) grades. As a result, the severity spectrum of cetuximab-associated skin adverse events cannot be reliably assessed in the present analysis. In addition, important clinical details, including exact onset time, cumulative drug exposure, concomitant medications, and detailed dermatological assessments, are often incomplete or missing, limiting causal inference. Moreover, disproportionality analyses are designed for signal detection rather than causal confirmation and should therefore be interpreted as hypothesis-generating.

Nevertheless, the use of two independent and complementary pharmacovigilance databases strengthens the robustness of our findings and reduces potential source-specific bias. Although spontaneous reporting data cannot replace prospective clinical studies, our results provide valuable real-world evidence that skin and subcutaneous tissue disorders represent the most prominent adverse events associated with cetuximab. Future well-designed observational studies, prospective cohorts, or dermatology-specific registries incorporating standardized severity assessments are warranted to better define incidence rates, severity distribution, underlying mechanisms, and long-term clinical implications.

## 5 Conclusion

Analyses of pharmacovigilance utilizing the FAERS and VigiAccess databases have revealed consistent disproportionality signals regarding cutaneous adverse events linked to cetuximab. Skin toxicities, notably including rash, acneiform dermatitis, erythema, acne, and dermatitis, were reported more frequently among middle-aged and older patients, particularly in males. Additionally, several events that were less commonly documented in clinical trials, such as exfoliative dermatitis, seborrheic dermatitis, and allergic dermatitis, have surfaced as significant safety signals in real-world settings. Although spontaneous reporting systems have inherent limitations, these results underscore the necessity for heightened clinical awareness. More prospective and mechanistic investigations are needed to validate these associations and elucidate the underlying mechanisms behind cetuximab-induced cutaneous toxicity.

## Data Availability

The original contributions presented in the study are included in the article/**Supplementary Material**. Further inquiries can be directed to the corresponding authors.
