# Supplementary material for: Characterization of skin adverse events associated with cetuximab: real-world insights from the two global pharmacovigilance databases of FAERS and VigiAccess
Source: Front Oncol. 2026 Feb 16;16:1768984. doi: 10.3389/fonc.2026.1768984 (PMC12950550; doi:10.3389/fonc.2026.1768984)
Supplement: Supplementary file 2 [file Table1.docx]

**Supplementary Table 1. Cetuximab-associated skin-related preferred terms with positive disproportionality signals in FAERS and Vigiaccess.**

| **Cetuximab** | **The report number** | **ROR (95%CI)** | **PRR(χ^2^)** | **EBGM (EBGM05)** | **IC (IC025)** |
| --- | --- | --- | --- | --- | --- |
| **FAERS** |  |  |  |  |  |
| Rash | 1841 | 4.68(4.47,4.91) | 4.56(5131.68) | 4.54(4.34) | 2.18(2.11) |
| Dermatitis acneiform | 596 | 125.64(115.36,136.85) | 124.31(65039.3) | 111.00(101.91) | 6.79(6.43) |
| Erythema | 442 | 2.34(2.13,2.57) | 2.33(336.20) | 2.33(2.12) | 1.22(1.08) |
| Dry skin | 346 | 3.02(2.72,3.36) | 3.01(463.26) | 3.00(2.70) | 1.59(1.42) |
| Acne | 290 | 4.16(3.71,4.67) | 4.15(690.51) | 4.13(3.68) | 2.05(1.86) |
| Skin reaction | 188 | 15.36(13.30,17.75) | 15.31(2479.02) | 15.10(13.08) | 3.92(3.60) |
| Skin fissures | 169 | 10.64(9.14,12.38) | 10.61(1456.01) | 10.51(9.03) | 3.39(3.09) |
| Skin disorder | 166 | 5.64(4.84,6.57) | 5.63(628.76) | 5.60(4.81) | 2.49(2.22) |
| Skin toxicity | 151 | 33.34(28.35,39.22) | 33.26(4576.43) | 32.24(27.41) | 5.01(4.50) |
| Dermatitis | 142 | 7.98(6.76,9.41) | 7.96(857.49) | 7.90(6.70) | 2.98(2.67) |
| Nail disorder | 92 | 12.81(10.43,15.74) | 12.79(988.14) | 12.65(10.30) | 3.66(3.19) |
| Palmar-plantar erythrodysaesthesia syndrome | 70 | 3.31(2.62,4.19) | 3.31(112.32) | 3.30(2.61) | 1.72(1.33) |
| Skin haemorrhage | 46 | 3.74(2.80,5.00) | 3.74(91.95) | 3.73(2.79) | 1.90(1.40) |
| Onychoclasis | 42 | 6.86(5.06,9.29) | 6.85(208.54) | 6.81(5.03) | 2.77(2.14) |
| Scab | 41 | 4.06(2.99,5.52) | 4.06(94.21) | 4.05(2.98) | 2.02(1.47) |
| Hair growth abnormal | 30 | 4.82(3.36,6.89) | 4.81(90.24) | 4.80(3.35) | 2.26(1.58) |
| Nail bed inflammation | 17 | 66.70(40.84,108.94) | 66.68(1032.77) | 62.68(38.37) | 5.97(3.12) |
| Skin necrosis | 17 | 3.65(2.27,5.88) | 3.65(32.64) | 3.64(2.26) | 1.87(0.99) |
| Cutaneous symptom | 15 | 26.22(15.71,43.78) | 26.21(354.75) | 25.59(15.33) | 4.68(2.60) |
| Exfoliative rash | 15 | 6.10(3.67,10.13) | 6.10(63.55) | 6.07(3.65) | 2.60(1.48) |
| Hypertrichosis | 13 | 7.57(4.39,13.07) | 7.57(73.63) | 7.53(4.36) | 2.91(1.58) |
| Onychomadesis | 13 | 3.98(2.31,6.87) | 3.98(28.93) | 3.97(2.30) | 1.99(0.94) |
| Nail discolouration | 13 | 3.71(2.15,6.40) | 3.71(25.64) | 3.70(2.15) | 1.89(0.86) |
| Onychalgia | 10 | 11.38(6.10,21.23) | 11.38(93.64) | 11.27(6.04) | 3.49(1.67) |
| Onycholysis | 9 | 6.64(3.45,12.78) | 6.63(42.79) | 6.60(3.43) | 2.72(1.16) |
| Rash follicular | 7 | 53.29(24.93,113.93) | 53.28(341.42) | 50.71(23.72) | 5.66(1.76) |
| Nail bed bleeding | 7 | 14.27(6.77,30.10) | 14.27(85.22) | 14.09(6.68) | 3.82(1.39) |
| Palmar erythema | 7 | 6.85(3.26,14.41) | 6.85(34.75) | 6.81(3.24) | 2.77(0.96) |
| Nail toxicity | 4 | 15.93(5.93,42.78) | 15.93(55.12) | 15.70(5.85) | 3.97(0.69) |
| Nail bed disorder | 4 | 10.43(3.90,27.94) | 10.43(33.77) | 10.34(3.86) | 3.37(0.55) |
| Nail bed tenderness | 3 | 28.81(9.15,90.76) | 4.56(5131.68) | 28.05(8.91) | 4.81(0.38) |
| **Vigiaccess** |  |  |  |  |  |
| Rash | 6232 | 3.38(3.30,3.47) | 3.25(9837.39) | 3.24(3.16) | 1.70(1.66) |
| Acne | 1996 | 20.59(19.69,21.53) | 20.23(35845.9) | 19.88(19.01) | 4.31(4.23) |
| Dermatitis acneiform | 1442 | 122.62(116.08,129.52) | 121.03(154199) | 108.81(103.01) | 6.77(6.58) |
| Dry skin | 1262 | 9.06(8.57,9.58) | 8.97(8872.33) | 8.90(8.42) | 3.15(3.06) |
| Skin fissures | 695 | 38.35(35.55,41.37) | 38.11(24256.1) | 36.84(34.14) | 5.20(5.02) |
| Skin reaction | 546 | 10.43(9.59,11.35) | 10.39(4589.13) | 10.30(9.46) | 3.36(3.22) |
| Skin exfoliation | 436 | 4.29(3.91,4.72) | 4.28(1092.88) | 4.27(3.88) | 2.09(1.94) |
| Skin toxicity | 436 | 68.27(61.96,75.23) | 68.01(27065.1) | 64.00(58.08) | 6.00(5.66) |
| Dermatitis | 286 | 4.81(4.28,5.40) | 4.80(856.24) | 4.78(4.26) | 2.26(2.07) |
| Skin disorder | 279 | 5.35(4.76,6.02) | 5.34(980.66) | 5.32(4.73) | 2.41(2.22) |
| Blister | 233 | 2.55(2.24,2.90) | 2.55(218.38) | 2.54(2.24) | 1.35(1.15) |
| Skin burning sensation | 218 | 2.59(2.26,2.95) | 2.58(211.02) | 2.58(2.26) | 1.37(1.16) |
| Nail disorder | 209 | 14.75(12.86,16.91) | 14.72(2637.20) | 14.54(12.68) | 3.86(3.57) |
| Skin lesion | 197 | 5.44(4.73,6.26) | 5.43(708.99) | 5.41(4.70) | 2.44(2.20) |
| Palmar-plantar erythrodysaesthesia syndrome | 164 | 4.48(3.84,5.22) | 4.47(440.61) | 4.46(3.82) | 2.16(1.90) |
| Pain of skin | 146 | 3.92(3.33,4.61) | 3.92(316.28) | 3.91(3.32) | 1.97(1.70) |
| Dermatitis allergic | 130 | 3.63(3.05,4.31) | 3.62(246.25) | 3.62(3.04) | 1.85(1.57) |
| Skin ulcer | 105 | 3.20(2.64,3.87) | 3.19(157.75) | 3.19(2.63) | 1.67(1.36) |
| Skin haemorrhage | 101 | 7.16(5.89,8.71) | 7.15(531.26) | 7.11(5.85) | 2.83(2.46) |
| Onychoclasis | 85 | 9.72(7.85,12.03) | 9.71(658.43) | 9.63(7.78) | 3.27(2.82) |
| Scab | 70 | 4.72(3.73,5.97) | 4.72(204.41) | 4.70(3.72) | 2.23(1.82) |
| Sensitive skin | 62 | 4.48(3.49,5.75) | 4.48(166.81) | 4.46(3.48) | 2.16(1.72) |
| Hair growth abnormal | 57 | 6.19(4.77,8.03) | 6.19(246.41) | 6.16(4.74) | 2.62(2.12) |
| Nail bed inflammation | 34 | 72.38(51.14,102.44) | 72.36(2240.85) | 67.83(47.92) | 6.08(4.04) |
| Skin atrophy | 30 | 4.00(2.80,5.73) | 4.00(67.33) | 3.99(2.79) | 2.00(1.34) |
| Xeroderma | 29 | 22.03(15.25,31.81) | 22.02(570.14) | 21.60(14.95) | 4.43(3.15) |
| Cutaneous symptom | 26 | 17.34(11.77,25.55) | 17.34(393.92) | 17.08(11.59) | 4.09(2.86) |
| Hypertrichosis | 26 | 5.05(3.44,7.43) | 5.05(84.11) | 5.03(3.42) | 2.33(1.57) |
| Skin wrinkling | 23 | 7.20(4.78,10.86) | 7.20(122.06) | 7.16(4.75) | 2.84(1.92) |
| Ingrowing nail | 23 | 9.95(6.60,15.00) | 9.94(183.32) | 9.86(6.54) | 3.30(2.26) |
| Exfoliative rash | 22 | 4.24(2.79,6.45) | 4.24(54.31) | 4.23(2.78) | 2.08(1.29) |
| Hyperkeratosis | 22 | 3.07(2.02,4.66) | 3.07(30.58) | 3.06(2.01) | 1.61(0.89) |
| Onychomadesis | 21 | 6.71(4.37,10.31) | 6.71(101.43) | 6.68(4.35) | 2.74(1.79) |
| Onychalgia | 19 | 16.85(10.71,26.51) | 16.85(278.79) | 16.60(10.55) | 4.05(2.57) |
| Onycholysis | 18 | 6.96(4.38,11.07) | 6.96(91.27) | 6.92(4.35) | 2.79(1.74) |
| Nail bed bleeding | 15 | 24.21(14.51,40.39) | 24.21(326.33) | 23.69(14.20) | 4.57(2.56) |
| Nail toxicity | 14 | 35.03(20.57,59.66) | 35.03(448.09) | 33.95(19.93) | 5.09(2.65) |
| Prurigo | 12 | 6.84(3.88,12.07) | 6.84(59.47) | 6.80(3.86) | 2.77(1.43) |
| Palmar erythema | 12 | 3.74(2.12,6.59) | 3.74(24.01) | 3.73(2.12) | 1.90(0.82) |
| Dermatosis | 10 | 6.92(3.72,12.89) | 6.92(50.31) | 6.88(3.69) | 2.78(1.29) |
| Rash follicular | 9 | 7.10(3.69,13.68) | 7.10(46.87) | 7.06(3.67) | 2.82(1.22) |
| Hangnail | 8 | 51.49(25.33,104.68) | 51.49(377.83) | 49.16(24.18) | 5.62(1.97) |
| Erythrosis | 8 | 23.42(11.62,47.18) | 23.42(167.98) | 22.93(11.38) | 4.52(1.76) |
| Nail cuticle fissure | 7 | 85.96(39.80,185.67) | 85.96(544.01) | 79.63(36.87) | 6.32(1.81) |
| Nail bed disorder | 6 | 13.73(6.14,30.71) | 13.73(69.89) | 13.56(6.06) | 3.76(1.18) |
| Nail ridging | 6 | 8.85(3.96,19.77) | 8.85(41.46) | 8.79(3.94) | 3.14(0.96) |
| Nail dystrophy | 5 | 4.90(2.04,11.80) | 4.90(15.45) | 4.88(2.03) | 2.29(0.39) |
| Subcutaneous emphysema | 5 | 5.88(2.44,14.15) | 5.88(20.12) | 5.85(2.43) | 2.55(0.51) |
| Nail fold inflammation | 4 | 74.97(27.20,206.63) | 74.97(272.79) | 70.12(25.44) | 6.13(0.90) |
| Fingerprint loss | 4 | 27.05(10.03,72.96) | 27.05(97.86) | 26.40(9.79) | 4.72(0.81) |
| Milia | 4 | 6.33(2.37,16.92) | 6.33(17.85) | 6.30(2.36) | 2.66(0.32) |
| Eczema asteatotic | 4 | 5.58(2.09,14.90) | 5.58(14.95) | 5.55(2.08) | 2.47(0.24) |
| Nail bed tenderness | 3 | 22.73(7.24,71.33) | 22.73(61.02) | 22.28(7.10) | 4.48(0.35) |
| Nail discomfort | 3 | 10.86(3.48,33.88) | 10.86(26.60) | 10.77(3.45) | 3.43(0.19) |
